# Draft Genome Sequences of 256 *Salmonella enterica* subsp. *enterica* Serovar Enteritidis Strains Isolated from Humans, Food, Chickens, and Farm Environments in Brazil

**DOI:** 10.1128/genomeA.00399-17

**Published:** 2017-07-13

**Authors:** Fábio Campioni, Guojie Cao, George Kastanis, Maria Sanchez Leon, Alzira Maria Morato Bergamini, Dália dos Prazeres Rodrigues, Marc William Allard, Juliana Pfrimer Falcão

**Affiliations:** a Departamento de Análises Clínicas, Toxicológicas e Bromatológicas, Faculdade de Ciências Farmacêuticas de Ribeirão Preto, Universidade de São Paulo, Ribeirão Preto, São Paulo, Brazil; b Division of Microbiology, Office of Regular Science, Center for Food Safety and Applied Nutrition, U.S. Food and Drug Administration, College Park, Maryland, USA; c Instituto Adolfo Lutz de Ribeirão Preto, Ribeirão Preto, São Paulo, Brazil; d Fundação Oswaldo Cruz, FIOCRUZ, Laboratório de Enterobactérias, Rio de Janeiro, Rio de Janeiro, Brazil

## Abstract

*Salmonella enterica* subsp. *enterica* serovar Enteritidis emerged in the late 1980s as the most isolated *Salmonella* serovar worldwide. Here, we report the draft genomes of 256 *S*. Enteritidis strains isolated from humans, food, chickens, and farm environments in Brazil. These draft genomes will help enhance our understanding of this serovar in Brazil.

## GENOME ANNOUNCEMENT

Salmonellosis caused by *Salmonella enterica* serovars has been a major health problem worldwide in terms of morbidity and mortality (1). *S. enterica* subsp. *enterica* serovar Enteritidis emerged in the late 1980s as one of the most isolated serovars from gastroenteritis cases linked to the consumption of raw or undercooked eggs and chickens in several countries (2, 3). In Brazil, the increase of *S*. Enteritidis isolation started in the late 1990s and currently remains one of the most isolated serovars in this country (4–7). Here, we announce 256 draft genome sequences from a collection of *S*. Enteritidis strains isolated in several states of Brazil from humans, food, chickens, and farm environments.

DNA from each strain was extracted according to Campioni et al. (8). Libraries were prepared using 1 ng of genomic DNA with the Nextera XT DNA library preparation kit (Illumina, San Diego, CA, USA), and the genomes were sequenced using the NextSeq 500 desktop sequencer with the NextSeq 500/500 high-output version 2 kit (300 cycles) (Illumina, San Diego, CA, USA) for 2 × 151 cycles according to the manufacturer’s instructions. *De novo* assemblies were generated from all raw sequence data. The Illumina reads were assembled with CLC Genomics Workbench version 9.5.2. The contigs for each isolate (draft genomes) were annotated using NCBI’s Prokaryotic Genome Annotation Pipeline (PGAP) (9). The genomes ranged between 4.6 and 4.8 Mb in size, as described for previous *Salmonella* isolates (4.6 Mb to almost 5.1 Mb) (10). The number of contigs per assembly for each isolate ranged from 52 to 183.

The data provided will help in understanding the epidemiology of *S*. Enteritidis strains isolated in Brazil from different sources. It will also provide phylogenetic insights into their evolution. A more detailed report of these genomic features will be addressed in a future publication.

### Accession number(s).

The draft genome sequences for the 256 *S*. Enteritidis isolates reported here are available in GenBank under the accession numbers listed in Table 1.

**TABLE 1:** Metadata for the 256 *S*. Enteritidis strains isolated from humans, food, chickens, and farm environments in Brazil

CFSAN^a^ no.	GenBank accession no.
CFSAN057625	MZNY00000000
CFSAN057626	MZNZ00000000
CFSAN057627	MZOA00000000
CFSAN057628	MZOB00000000
CFSAN057629	MZOC00000000
CFSAN057630	MZOD00000000
CFSAN057631	MZOE00000000
CFSAN057632	MZOF00000000
CFSAN057633	MZOG00000000
CFSAN057634	MZOH00000000
CFSAN057635	MZOI00000000
CFSAN057636	MZOJ00000000
CFSAN057637	MZOK00000000
CFSAN057638	MZOL00000000
CFSAN057639	MZOM00000000
CFSAN057640	MZON00000000
CFSAN057641	MZOO00000000
CFSAN057642	MZOP00000000
CFSAN057643	MZOQ00000000
CFSAN057644	MZOR00000000
CFSAN057645	MZOS00000000
CFSAN057646	MZOT00000000
CFSAN057647	MZOU00000000
CFSAN057648	MZOV00000000
CFSAN057649	MZOW00000000
CFSAN057650	MZOX00000000
CFSAN057651	MZOY00000000
CFSAN057652	MZOZ00000000
CFSAN057653	MZPA00000000
CFSAN057654	MZPB00000000
CFSAN057655	MZPC00000000
CFSAN057656	MZPD00000000
CFSAN057657	MZPE00000000
CFSAN057658	MZPF00000000
CFSAN057659	MZPG00000000
CFSAN057660	MZPH00000000
CFSAN057661	MZPI00000000
CFSAN057662	MZPJ00000000
CFSAN057663	MZPK00000000
CFSAN057664	MZPL00000000
CFSAN057665	MZPM00000000
CFSAN057666	MZPN00000000
CFSAN057667	MZPO00000000
CFSAN057668	MZPP00000000
CFSAN057669	MZPQ00000000
CFSAN057670	MZPR00000000
CFSAN057671	MZPS00000000
CFSAN057672	MZPT00000000
CFSAN057673	MZPU00000000
CFSAN057674	MZPV00000000
CFSAN057675	MZPW00000000
CFSAN057676	MZPX00000000
CFSAN057677	MZPY00000000
CFSAN057678	MZPZ00000000
CFSAN057679	MZQA00000000
CFSAN057680	MZQB00000000
CFSAN057681	MZQC00000000
CFSAN057682	MZQD00000000
CFSAN057683	MZQE00000000
CFSAN057684	MZQF00000000
CFSAN057685	MZQG00000000
CFSAN057686	MZQH00000000
CFSAN057687	MZQI00000000
CFSAN057688	MZQJ00000000
CFSAN057689	MZQK00000000
CFSAN057690	MZQL00000000
CFSAN057691	MZQM00000000
CFSAN057692	MZQN00000000
CFSAN057693	MZQO00000000
CFSAN057694	MZQP00000000
CFSAN057695	MZQQ00000000
CFSAN057696	MZQR00000000
CFSAN057697	MZQS00000000
CFSAN057698	MZQT00000000
CFSAN057699	MZQU00000000
CFSAN057700	MZQV00000000
CFSAN057701	MZQW00000000
CFSAN057702	MZQX00000000
CFSAN057703	MZQY00000000
CFSAN057704	MZQZ00000000
CFSAN057705	MZRA00000000
CFSAN057706	MZRB00000000
CFSAN057707	MZRC00000000
CFSAN057708	MZRD00000000
CFSAN057709	MZRE00000000
CFSAN057710	MZRF00000000
CFSAN057711	MZRG00000000
CFSAN057712	MZRH00000000
CFSAN057713	MZRI00000000
CFSAN057714	MZRJ00000000
CFSAN057715	MZRK00000000
CFSAN057716	MZRL00000000
CFSAN057717	MZRM00000000
CFSAN057718	MZRN00000000
CFSAN057719	MZRO00000000
CFSAN057720	MZRP00000000
CFSAN057721	MZRQ00000000
CFSAN057722	MZRR00000000
CFSAN057723	MZRS00000000
CFSAN057724	MZRT00000000
CFSAN057725	MZRU00000000
CFSAN057726	MZRV00000000
CFSAN057727	MZRW00000000
CFSAN057728	MZRX00000000
CFSAN057729	MZRY00000000
CFSAN057730	MZRZ00000000
CFSAN057731	MZSA00000000
CFSAN057732	MZSB00000000
CFSAN057733	MZSC00000000
CFSAN057734	MZSD00000000
CFSAN057735	MZSE00000000
CFSAN057736	MZSF00000000
CFSAN057737	MZSG00000000
CFSAN057738	MZSH00000000
CFSAN057739	MZSI00000000
CFSAN057740	MZSJ00000000
CFSAN057741	MZSK00000000
CFSAN057742	MZSL00000000
CFSAN057743	MZSM00000000
CFSAN057744	NBMZ00000000
CFSAN057745	MZSN00000000
CFSAN057746	MZSO00000000
CFSAN057747	MZSP00000000
CFSAN057748	MZSQ00000000
CFSAN057749	MZSR00000000
CFSAN057750	MZSS00000000
CFSAN057751	MZST00000000
CFSAN057752	MZSU00000000
CFSAN057753	MZSV00000000
CFSAN057754	MZSW00000000
CFSAN057755	MZSX00000000
CFSAN057756	MZSY00000000
CFSAN057757	MZSZ00000000
CFSAN057758	MZTA00000000
CFSAN057759	MZTB00000000
CFSAN057760	MZTC00000000
CFSAN057761	MZTD00000000
CFSAN057762	MZTE00000000
CFSAN057763	MZTF00000000
CFSAN057764	MZTG00000000
CFSAN057765	MZTH00000000
CFSAN057766	MZTI00000000
CFSAN057767	MZTJ00000000
CFSAN057768	MZTK00000000
CFSAN057769	MZTL00000000
CFSAN057770	MZTM00000000
CFSAN057771	MZTN00000000
CFSAN057772	MZTO00000000
CFSAN057773	NBNC00000000
CFSAN057774	MZTP00000000
CFSAN057775	MZTQ00000000
CFSAN057776	MZTR00000000
CFSAN057777	MZTS00000000
CFSAN057778	MZTT00000000
CFSAN057779	MZTU00000000
CFSAN057780	MZTV00000000
CFSAN057781	MZTW00000000
CFSAN057782	MZTX00000000
CFSAN057783	MZTY00000000
CFSAN057784	MZTZ00000000
CFSAN057785	MZUA00000000
CFSAN057786	MZUB00000000
CFSAN057787	MZUC00000000
CFSAN057788	MZUD00000000
CFSAN057789	MZUE00000000
CFSAN057790	MZUF00000000
CFSAN057791	MZUG00000000
CFSAN057792	MZUH00000000
CFSAN057793	MZUI00000000
CFSAN057794	MZUJ00000000
CFSAN057795	MZUK00000000
CFSAN057796	MZUL00000000
CFSAN057797	MZUM00000000
CFSAN057798	MZUN00000000
CFSAN057799	MZUO00000000
CFSAN057800	MZUP00000000
CFSAN057801	MZUQ00000000
CFSAN057802	MZUR00000000
CFSAN057803	MZUS00000000
CFSAN057804	MZUT00000000
CFSAN057805	MZUU00000000
CFSAN057806	MZUV00000000
CFSAN057807	MZUW00000000
CFSAN057808	MZUX00000000
CFSAN057809	MZUY00000000
CFSAN057810	MZUZ00000000
CFSAN057811	MZVA00000000
CFSAN057812	MZVB00000000
CFSAN057813	MZVC00000000
CFSAN057814	MZVD00000000
CFSAN057815	MZVE00000000
CFSAN057816	MZVF00000000
CFSAN057817	MZVG00000000
CFSAN057818	MZVH00000000
CFSAN057819	MZVI00000000
CFSAN057820	MZVJ00000000
CFSAN057821	MZVK00000000
CFSAN057823	MZVL00000000
CFSAN057825	MZVM00000000
CFSAN057826	MZVN00000000
CFSAN057827	MZVO00000000
CFSAN057828	MZVP00000000
CFSAN057829	MZVQ00000000
CFSAN057830	MZVR00000000
CFSAN057831	MZVS00000000
CFSAN057832	MZVT00000000
CFSAN057833	MZVU00000000
CFSAN057834	NBNB00000000
CFSAN057835	MZVV00000000
CFSAN057836	MZVW00000000
CFSAN057837	MZVX00000000
CFSAN057838	MZVY00000000
CFSAN057839	MZVZ00000000
CFSAN057840	MZWA00000000
CFSAN057841	MZWB00000000
CFSAN057842	MZWC00000000
CFSAN057843	MZWD00000000
CFSAN057844	MZWE00000000
CFSAN057845	MZWF00000000
CFSAN057846	MZWG00000000
CFSAN057847	MZWH00000000
CFSAN057848	MZWI00000000
CFSAN057849	MZWJ00000000
CFSAN057850	MZWK00000000
CFSAN057851	MZWL00000000
CFSAN057852	MZWM00000000
CFSAN057853	MZWN00000000
CFSAN057854	MZWO00000000
CFSAN057855	MZWP00000000
CFSAN057858	MZWQ00000000
CFSAN057859	MZWR00000000
CFSAN057860	MZWS00000000
CFSAN057861	MZWT00000000
CFSAN057862	MZWU00000000
CFSAN057863	MZWV00000000
CFSAN057864	MZWW00000000
CFSAN057865	MZWX00000000
CFSAN057866	NBNA00000000
CFSAN057867	MZWY00000000
CFSAN057868	MZWZ00000000
CFSAN057869	MZXA00000000
CFSAN057870	MZXB00000000
CFSAN057872	MZXC00000000
CFSAN057873	MZXD00000000
CFSAN057874	MZXE00000000
CFSAN057875	MZXF00000000
CFSAN057880	MZXG00000000
CFSAN057881	MZXH00000000
CFSAN057882	MZXI00000000
CFSAN057883	MZXJ00000000
CFSAN057884	MZXK00000000
CFSAN057885	MZXL00000000
CFSAN057888	MZXM00000000
CFSAN057889	MZXN00000000
CFSAN057890	MZXO00000000
CFSAN057891	MZXP00000000

a CFSAN, Center for Food Safety and Applied Nutrition (FDA).
